# Sirt3 Attenuates Oxidative Stress Damage and Rescues Cellular Senescence in Rat Bone Marrow Mesenchymal Stem Cells by Targeting Superoxide Dismutase 2

**DOI:** 10.3389/fcell.2020.599376

**Published:** 2020-11-16

**Authors:** Cao Ma, Yanan Sun, Chenchen Pi, Huan Wang, Hui Sun, Xiao Yu, Yingai Shi, Xu He

**Affiliations:** ^1^The Key Laboratory of Pathobiology, Ministry of Education, College of Basic Medical Sciences, Jilin University, Changchun, China; ^2^Department of Pathology, Zhongda Hospital, School of Medicine, Southeast University, Nanjing, China; ^3^The First Hospital, Institute of Immunology, Jilin University, Changchun, China

**Keywords:** mesenchymal stem cells, Sirt3, oxidative stress, cellular senescence, superoxide dismutase 2

## Abstract

Oxidative stress is one of the main causes of aging. The process of physiological aging is always accompanied by increased levels of endogenous oxidative stress. Exogenous oxidants have contributed to premature cellular senescence. As a deacetylase located in mitochondrial matrix, Sirt3 plays critical roles in mitochondrial energy metabolism, oxidative stress regulation, and cellular senescence. However, it remains unknown whether Sirt3 exerts the analogous role in cellular senescence caused by two different oxidation pathways. In this study, the function of Sirt3 was investigated in age-related natural senescence and H_2_O_2_-induced premature senescence of rat bone marrow mesenchymal stem cells (MSCs). Our results showed that Sirt3 expression was significantly decreased in both senescent MSCs, which was concerned with reduced cellular reactive oxygen species (ROS) and aggravated DNA injury. Sirt3 repletion could partly reverse the senescence-associated phenotypic features in natural and premature senescent MSCs. Moreover, Sirt3 replenishment led to the reduction in the levels of cellular ROS by enhancing the expression and activity of superoxide dismutase 2 (SOD2), thus maintaining the balance of intracellular oxidation and antioxidation and ameliorating oxidative stress damage. Altogether, Sirt3 inhibits MSC natural senescence and H_2_O_2_-induced premature senescence through alleviating ROS-induced injury and upregulating SOD2 expression and activity. Our research indicates that Sirt3 might contribute to uncovering the novel mechanisms underlying MSC senescence and provide new insights to aging and oxidative stress-related diseases.

## Introduction

Establishment of aging models is an important method to investigate the mechanisms of human aging and to exploit anti-aging drugs, including cellular senescence models and animal aging models (Hayflick and Moorhead, 1961; von Zglinicki et al., 1995; Mitchell et al., 2015). However, each model has its own limitations, and thus, they are utilized in different applications (Folch et al., 2018). Natural aging model is one of the most common animal models, from which cells extracted can simulate the normal aging process and are the closest to physiological aging (Ikeda, 2011). In the aging process, the organism is inevitably exposed to various damaging factors, among which oxidative stress is one of the most common reasons of aging (Brandl et al., 2011). Our previous research found that mesenchymal stem cells obtained from aged rats generated more excessive reactive oxygen species (ROS) than cells from young rats, indicating that endogenous ROS accumulated in cells with increasing age (Ma et al., 2017). Precocious senescence is characterized by the addition of exogenous stimuli that lead to cellular premature senescence. The numerous stimulants are easy to obtain, cheap, and non-toxic, which can effectively induce cellular senescence. Accumulating studies have proved that exogenous oxidants can induce cellular premature senescence. Owing to safety, non-toxicity, and easy obtainment, hydrogen peroxide (H_2_O_2_) is widely used in the establishment of premature senescence model (Ko et al., 2012). The essence of individual aging is cellular senescence (López-Otín et al., 2013). The process of individual aging is accompanied by a dramatic elevation in ROS level, and multiple age-related diseases are closely associated with the alteration of oxidative stress, such as Alzheimer’s disease, chronic obstructive pulmonary disease, type 2 diabetes, and age-related hearing loss (Zeng et al., 2014; Conti et al., 2015; Lemos et al., 2017; Wojsiat et al., 2018). Hence, potential targets for anti-oxidation to delay or reverse cellular senescence and individual aging are worth exploring.

In mammals, there are seven members of sirtuin family, sirt1–sirt7, with distinct subcellular localizations and functions. Sirt3 is one of the deacetylases located in the mitochondria, which can regulate the activity of multiple enzymes through deacetylation, thus affecting mitochondrial function and cellular physiological condition (Finkel et al., 2009). It has been confirmed that Sirt3 plays a critical role in the elimination of intracellular ROS and maintenance of oxygen metabolism balance (Yu et al., 2016; Denu, 2017). Growing evidences have indicated that Sirt3/superoxide dismutase 2 (SOD2) pathway is closely related to aging (Wang et al., 2014; Zhou et al., 2020). Recent studies have suggested that SOD2 is a specific target of Sirt3. Sirt3 effectively promotes SOD2 activity through the deacetylation of lysine 53, 68, and 89 (Qiu et al., 2010; Gao et al., 2018). Sirt3 over-expression in porcine fetal fibroblasts (PFF) can slow down cellular senescence by attenuating DNA damage (Xie et al., 2017). In a study of age-related hearing loss, mice lacking Sirt3 (Sirt3^–/–^) significantly lose their protective role against oxidative damage compared to wild-type mice (Someya et al., 2010), manifesting that Sirt3-mediated mitochondrial oxygen metabolism may be a pivotal regulatory mechanism of aging retardation.

Mesenchymal stem cells (MSCs) are a kind of multipotent stem cells and are mainly derived from the bone marrow, fat, cord blood, and placenta (Friedenstein et al., 1970). MSCs function as precursors to a variety of cell types, including adipocytes, osteoblasts, and chondrocytes. Due to easier and safer obtainment and lower immunogenicity, MSCs have been overwhelmingly useful seed cells in tissue engineering and regenerative medicine. However, as individuals age and oxidative stress injury accumulates or removes abnormally, MSCs also exhibit senescence-like characteristics. Thereby, whether Sirt3 can rejuvenate senescent MSCs and its mechanisms merits urgent investigation. In our previous study, we revealed that ROS levels of MSCs from naturally aged rats were significantly higher than those of young individuals. In addition, we also verified an age-dependent decrease in NAD^+^ content. In view of the intimate connection between Sirt3 and oxidative metabolism, as well as its biological characteristics of NAD^+^ dependence, we speculate that Sirt3 might exert a certain influence on MSC senescence by manipulating the cellular oxidation levels. In order to further explore the interaction between Sirt3 and oxidative stress in MSC senescence, exogenous oxidant H_2_O_2_ was added to enhance cellular ROS levels and promote premature senescence. Then age-related alterations in morphology and senescence-related markers, including cell proliferation, apoptosis, senescence-associated-β-galactosidase (SA-β-gal) activity, p16^INK4A^, and p21^WAF1/CIP1^ expression, were detected. Moreover, we evaluated the levels of cellular ROS, the expression and activity of SOD2, malondialdehyde (MDA) contents, and DNA damage markers to identify the levels of intracellular oxidative stress in natural senescent and H_2_O_2_-induced senescent MSCs. In addition, senescent MSCs were transduced with lentivirus carrying Sirt3 to elucidate Sirt3 effects on cellular senescence and oxidative metabolism.

## Results

### Senescence Induction and Age-Associated Variations in Mesenchymal Stem Cells

**FIGURE 1:**
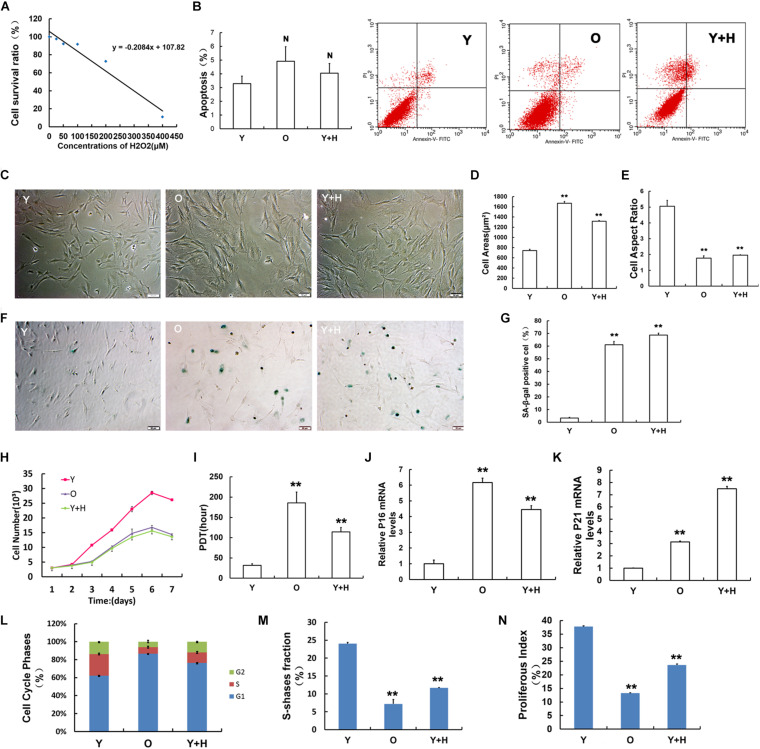
Obtainment of senescent mesenchymal stem cells (MSCs) and identification of senescence-associated characteristics. **(A)** Cell viability of MSCs after 2 h treatment with H_2_O_2_ at different concentrations evaluated by CCK-8 assay. **(B)** Cell apoptosis assays in young (Y), old (O), and H_2_O_2_ pretreated (Y + H) MSCs. **(C)** Age-related changes in cell morphology. **(D)** Cell surface areas. **(E)** Cell aspect ratio. **(F)** Senescence-associated-β-galactosidase (SA-β-gal) staining. **(G)** Quantitative analysis of SA-β-gal staining. **(H)** Cell growth curves. **(I)** Population doubling time (PDT). mRNA expression of the senescence-related factor **(J)** pl6^INK4A^ and **(K)** p21^WAF1/CIP1^. **(L)** Cell cycle analysis. **(M)** Fraction of cells in S-phase (SPF). **(N)** Proliferation index (PI). Data are expressed as mean ± SD, *n* = 3. **P < 0.01, **^N^***P* > 0.05 vs. young.

Premature senescence in young MSCs (Y) was established by exposure to H_2_O_2_ at a sub-lethal concentration as reported in a previous literature (Kumar et al., 2019). First, the optimal concentration of H_2_O_2_-induced premature senescence without obvious cytotoxicity was assessed by CCK-8 method (Figure 1A). Then, H_2_O_2_ at the concentration of 250 μM was selected as the exogenous oxidant to induce young MSC senescence in subsequent experiments (recorded as Y + H). Natural senescent MSCs (O) were extracted from15 to 18 months-old rats, as previously described (Ma et al., 2017). In order to eliminate the apoptotic interference, cell apoptosis was detected by flow cytometry. The results displayed that there was no significant difference among the three groups (Figure 1B). After treatment with H_2_O_2_, MSCs obtained from young rats showed senescence-like morphology with irregular shapes, flattened and enlarged cell bodies, and attenuated stereoscopic perception (Figure 1C), similar to the natural senescent MSCs. Statistical analysis of cell morphology revealed that the cell aspect ratios markedly decreased, whereas cell areas significantly increased both in natural senescent and premature senescent MSCs compared with young group (Figures 1D,E). SA-β-gal staining is considered as the classical standard for evaluation of cellular senescence (Dimri et al., 1995). The number of SA-β-gal-positive blue cells in natural senescent MSCs was extraordinarily higher than that in young MSCs. After H_2_O_2_ treatment, blue-stained cells in young MSCs also remarkably augmented, suggesting cellular premature senescence occurred (Figures 1F,G). The cell growth curves were drawn to observe cell proliferation. Data analysis results demonstrated that in young MSCs treated with H_2_O_2_, as well as the natural senescent MSCs, there was a decline in cell proliferation (Figure 1H). Moreover, the population doubling time (PDT) in both cells was prolonged to 6 and 3.6 times, respectively (Figure 1I). Pl6^INK4A^ and p21^WAF1/CIP1^, as the acknowledged biological indicators, were widely used in the assessment of cellular senescence (Choudhery et al., 2014). To evaluate senescence-associated alterations at molecular levels, mRNA expression of pl6^INK4A^ and p21^WAF1/CIP1^ were further monitored. As indicated in Figure 1J, pl6^INK4A^ expression levels were heightened in natural senescent MSCs, as well as H_2_O_2_-treated young MSCs. Similar trends could also be observed in the expression levels of p21^WAF1/CIP1^ (Figure 1K). Cell cycle analysis revealed that more cells stuck in G1 phase, whether elevated endogenous oxidation in old group or addition of exogenous oxidant in H_2_O_2_ group (Figure 1L). In addition, the S-phase fraction (SPF) and proliferative index (PI) were lower in both senescent cells than those in the young group (Figures 1M,N).

### Reduced Antioxidant Capacity and Aggravated DNA Injury in Senescent MSCs Is Associated With Down-Regulated Sirt3 Expression via Attenuated SOD2 Expression and Activity

**FIGURE 2:**
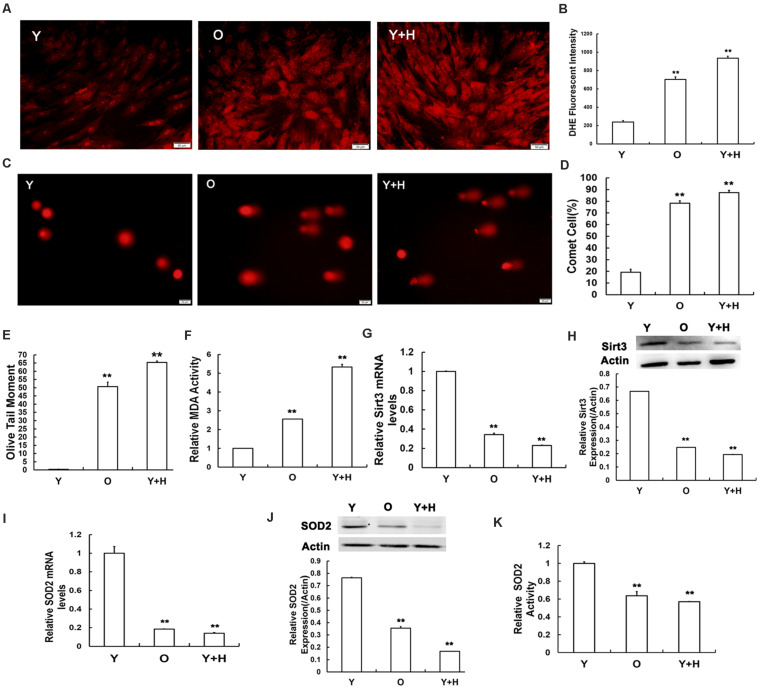
Senescence-relevant alterations in oxidation/antioxidation capacity and measurement of Sirt3 and superoxide dismutase 2 (SOD2) expression and activity. **(A)** Intracellular reactive oxygen species (ROS) levels were determined using dihydroethidium (DHE) staining in young (Y), old (O), and H_2_O_2_ pretreated (Y + H) MSCs. **(B)** Quantitative analysis of DHE-fluorescent intensity. **(C–E)** DNA damage was detected by comet assays **(C)**, quantitative analysis of comet cells ratio **(D)**, and measurement of olive tail moments (OTMs) **(E)**. **(F)** Intracellular malondialdehyde (MDA) contents. **(G)** Sirt3 mRNA expression was detected by real-time quantitative polymerase chain reaction (RT-qPCR). **(H)** Sirt3 protein expression was detected by western blotting. **(I)** SOD2 mRNA expression was examined by RT-qPCR. **(J)** SOD2 protein expression was examined by western blotting. **(K)** Assessment of SOD2 activity. Data are expressed as mean ± SD, *n* = 3. **P < 0.01 vs. young.

In our previous study, we demonstrated that ROS accumulation in MSCs obtained from chronological aged rats. To further investigate the effects of oxidative stress, MSCs from young rats were treated with exogenous oxidant H_2_O_2_ at the concentration of 250 μM to increase the level of cellular ROS. Dihydroethidium (DHE) staining and flow cytometry were then performed to determine intracellular ROS levels. As shown in Figure 2A, the intensity of ROS fluorescence was much weaker in the young MSCs than that in old or H_2_O_2_-treated cells. The quantitative analysis results of flow cytometry illustrated that intracellular ROS level in the young group was up-regulated by approximately fivefold after H_2_O_2_ treatment (Figure 2B). ROS can trigger severe damage to cellular macromolecules, particularly making them prone to DNA damage (Cooke et al., 2003; Barzilai and Yamamoto, 2004). Therefore, a single-cell gel electrophoresis assay was further carried out to evaluate the extent of DNA breaks (Figure 2C). Quantitative analysis indicated the ratio of injured MSCs increased (Figure 2D), and the length of olive tail moment (OTM) (Figure 2E) prolonged in the old group and H_2_O_2_ group compared with the young control. Next, we also assessed intracellular MDA contents, a biomarker of lipid peroxidation in living cells (Lin et al., 2019). The data showed that intracellular MDA production was elevated along with ROS accumulation under chronologic aging conditions, as well as after the addition of exogenous oxidant (Figure 2F).

To explore the potential roles of Sirt3 in ROS-correlated senescence, we detected Sirt3 expression by real-time quantitative polymerase chain reaction (RT-qPCR) and western blotting in all three groups. At both mRNA (Figure 2G) and protein (Figure 2H) levels, Sirt3 expression was significantly diminished due to ROS accumulation caused by endogenous (old group) or exogenous oxidation (H_2_O_2_ group). Ample evidence displayed that SOD2 might be a potential specific target of Sirt3 (Chen et al., 2011; He et al., 2019). To verify whether Sirt3 was involved in the regulation of ROS-related senescence via SOD2 mediation, we examined the alterations of SOD2 expression and activity. The mRNA levels of SOD2 expression were both lower in natural senescent and premature senescent MSCs than those in the young group by 5.56- and 7.14-fold, respectively (Figure 2I), and similar downtrends were found in SOD2 protein expression. The expression levels of Sirt3 protein in old MSCs or H_2_O_2_-treated MSCs were 2.86- and 6.25-fold lower than those in the young counterparts (Figure 2J). In addition, various degrees of suppression in Sirt3 activity were observed in both senescent MSCs (Figure 2K).

### Sirt3 Replenishment Accelerates Clearance of Intracellular Excessive ROS

**FIGURE 3:**
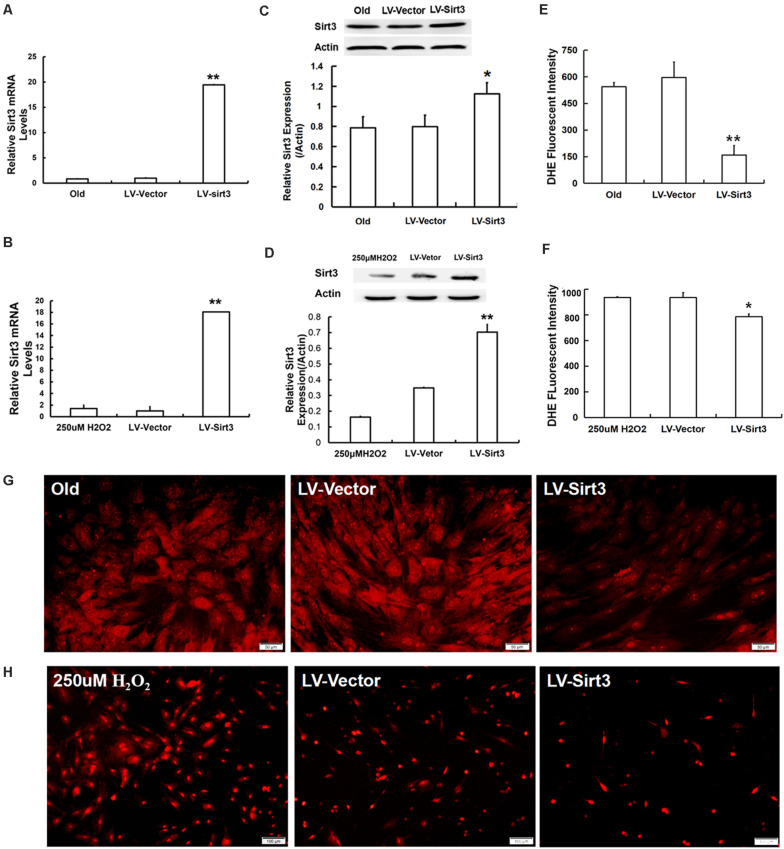
Sirt3 replenishment improves ROS scavenging ability. **(A,B)** Sirt3 mRNA expression was determined by RT-qPCR in old MSCs **(A)** and H_2_O_2_-pretreated MSCs **(B)** after Sirt3 over-expression. **(C,D)** Sirt3 protein expression was tested by western blotting. **(E–H)** DHE fluorescence intensity was detected by flow cytometry and microphotography. Data are expressed as mean ± SD, *n* = 3. *P < 0.05, **P < 0.01 vs. old or H_2_O_2_.

To further explore the roles of Sirt3 in modulating oxidative stress, old and H_2_O_2_-treated MSCs were, respectively, transduced with lentivirus-expressing Sirt3 (LV-Sirt3) and the lentiviral vector (LV-Vector), followed by evaluating transduction efficiency though RT-qPCR and western blotting. The results confirmed that Sirt3 was successfully up-regulated at both mRNA (Figures 3A,B) and protein (Figures 3C,D) levels. Subsequently, intracellular ROS was measured in both LV-Sirt3 group and LV-Vector group. As demonstrated in Figures 3E,F, ROS amassing was notably diminished in Sirt3-replenished cells as compared to the control (LV-Vector), no matter in natural senescent MSCs or premature senescent MSCs. The results of DHE fluorescent staining were consistent with those of flow cytometry (Figures 3G,H).

### Sirt3 Over-Expression Alleviates ROS-Relevant MSC Senescence

**FIGURE 4:**
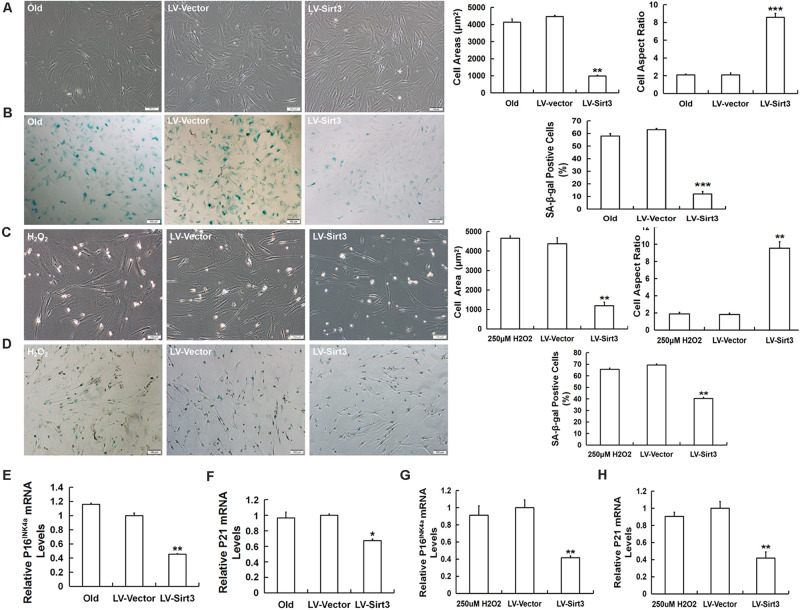
Sirt3 over-expression mitigates senescence-associated phenotypic features in natural senescent MSCs and H_2_O_2_-induced premature senescent MSCs. **(A)** After Sirt3 over-expression, age-related morphological alterations were evaluated in both senescent MSCs, and the cell areas and the cell aspect ratio were quantified. **(B)** SA-β-gal staining and quantification were performed. Meanwhile, lentivirus-expressing Sirt3 (LV-Sirt3) was also transduced into H_2_O_2_-treated MSCs, and its morphological alterations **(C)** and the ratio of SA-β-gal-positive cells **(D)** were assessed. Gene expression of senescence-related factor pl6^INK4A^ **(E)** and p21^WAF1/CIP1^ **(F)** in MSCs obtained from old rats after Sirt3 repletion. Gene expression of senescence-related factor pl6^INK4A^ **(G)** and p21^WAF1/CIP1^ **(H)** in Sirt3 over-expressed MSCs derived from H_2_O_2_ treatment. Data are expressed as mean ± SD, *n* = 3. *P < 0.05, **P < 0.01, ***P < 0.001 vs. old or H_2_O_2_.

To investigate whether Sirt3 directly influenced MSC senescence, we examined the morphological characteristics and senescent biomarkers in senescent MSCs after Sirt3 over-expression. Old MSCs after Sirt3 replenishment exhibited ameliorated senescent morphology with long and fusiform shape, enhanced the stereoscopic perception, declined cell surface area, and increased cell aspect ratio (Figure 4A). Moreover, the number of senescence-associated blue-stained cells markedly decreased following Sirt3 over-expression (Figure 4B). In accordance with the old group, Sirt3 repletion improved large and flat cellular morphology caused by H_2_O_2_ stimulation. Statistical data displayed that the cell aspect ratio was dramatically augmented, whereas the cell surface area was diminished after Sirt3 repletion (Figure 4C). In addition, SA-β-gal activity in Sirt3-overexpressed MSCs was largely abated compared to that in cells transduced with the vector (Figure 4D). Alterations at the molecular levels that resulted from Sirt3 sufficiency have also been unraveled in natural senescent and premature senescent cells. Both pl6^INK4A^ and p21^WAF1/CIP1^ mRNA levels were evaluated by using RT-qPCR. Contrary to Sirt3 up-regulation, the expression of pl6^INK4A^ and p21^WAF1/CIP1^ was obviously reduced in the LV-Sirt3 group compared to the vector group (Figures 4E–H), indicating senescence-associated genetic indexes can be effectively rescued as a result of Sirt3 replenishment.

### Sirt3 Attenuated MSC Senescence via Enhancing SOD2 Activation and Reducing Oxidative Stress Damage

**FIGURE 5:**
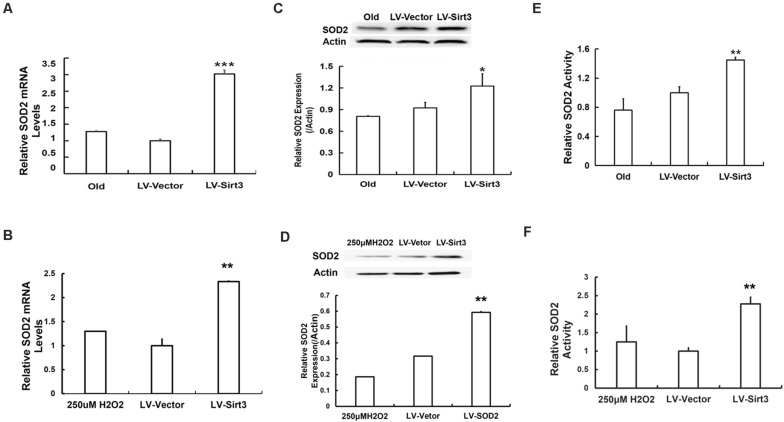
Sirt3 repletion slows down MSC senescence associated with enhanced SOD2 expression and activity. SOD2 mRNA levels were tested in old **(A)** and H_2_O_2_-treated **(B)** MSCs after Sirt3 transduction. Protein levels of SOD2 **(C,D)** were measured using western blotting as well. SOD2 activity was also evaluated in old **(E)** or H_2_O_2_-treated **(F)** cells after Sirt3 over-expression. Data are expressed as mean ± SD, *n* = 3. *P < 0.05, **P < 0.01, ***P < 0.001 vs. old or H_2_O_2_.

To further explore the possible mechanisms of Sirt3-regulated MSC senescence, we then examined intracellular SOD2 level and its activity. The results presented that SOD2 was remarkably up-regulated at the mRNA level in Sirt3-sufficient MSCs (Figures 5A,B). Additionally, SOD2 protein expression in Sirt3-overexpressed cells from old or H_2_O_2_-treated MSCs showed a semblable upward trend (Figures 5C,D). Furthermore, we examined whether SOD2 activity was enhanced in response to Sirt3 supplement. The data showed that Sirt3 over-expression contributed to the maintenance of SOD2 activity not only in old MSCs (Figure 5E) but also in H_2_O_2_-exposed MSCs (Figure 5F). The results indicated that the molecular mechanism underlying Sirt3 ameliorating MSC senescence is associated with elevated SOD2 expression and activity.

**FIGURE 6:**
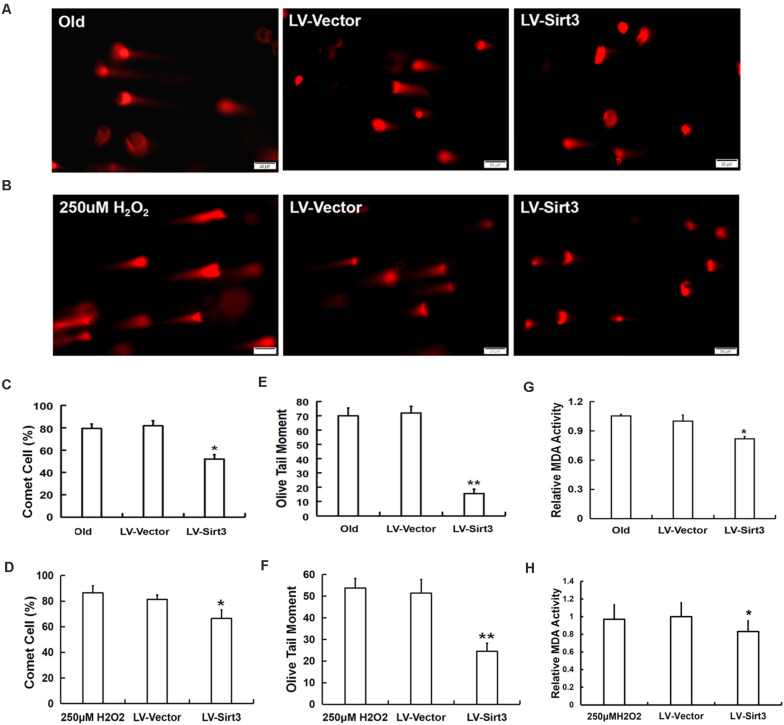
Sirt3 alleviated MSC senescence through ameliorating DNA damage and intracellular MDA contents. DNA damage was determined by comet assay in both natural and premature senescent cells upon Sirt3 replenishment **(A,B)**. Fluorescent images and the quantitative analysis of comet-positive cells **(C,D)** and olive tail moments (OTM) **(E,F)**. **(G,H)** The levels of intracellular MDA were determined by colorimetry in the old group **(G)** and H_2_O_2_-treated group **(H)**. Data are expressed as mean ± SD, *n* = 3. *P < 0.05, **P < 0.01 vs. old or H_2_O_2_.

To confirm whether elevated SOD2 activation mediated by Sirt3 repletion played important roles in ROS-relevant senescence and oxidative stress injury, DNA damage and MDA levels were further examined following Sirt3 over-expression in senescent MSCs. Sirt3 abundance either in old cells or in H_2_O_2_-pretreated cells significantly attenuated DNA damage (Figures 6A–F) and intracellular MDA contents (Figures 6G,H) compared to that in their own LV-vector group.

## Discussion

Aging is a complex phenomenon, and emerging scientific researches have brought up different theories trying to reveal its mystery. Among which, the free radical theory of aging was one of the most widely accepted theories proposed by Harman (1956). On this basis, the theory of oxidative stress aging has been developed and gradually gained considerable acceptance (Grune et al., 2005; Liguori et al., 2018). In simple terms, the cumulative damages induced by high levels of endogenous/exogenous ROS are detrimental factors for the functional maintenance of biological macromolecules such as DNA, lipids, and proteins (Schieber and Chandel, 2014). Excessive ROS mediated protein denaturation, lipid peroxidation, DNA modification, and mitochondrial dysfunction and ultimately led to cellular senescence (Chen et al., 2017). In fact, when exogenous oxide (H_2_O_2_) was added, different types of young cells transformed into senescent phenotypes (Ben-Porath and Weinberg, 2005). In addition, endogenously formed ROS, such as superoxide (O_2_^–^) and the highly reactive hydroxyl radical (^–^OH), also played a crucial role in the process of cellular senescence (Höhn et al., 2017). Although several key pathways are leading to oxidative stress-related stem cell senescence, our study focused on the sirtuin pathway.

The previous studies indicated that cell proliferation slowed down, the PDT increased, osteogenic and adipogenic differentiation potentials diminished, and cell cycle arrested in senescent MSCs (Ma et al., 2017; Pi et al., 2019), consistent with our present results. Importantly, MSC senescence has contributed to tissue, organ, and organism aging and age-related diseases. Macrophage migration inhibitory factor (MIF) can rejuvenate senescent MSCs by activating autophagy and enhancing their therapeutic efficacy for myocardial infarction (Zhang et al., 2019). It has been demonstrated that only young MSC-derived extracellular vesicles (MSC-EVs) are able to alleviate lipopolysaccharide (LPS)-induced acute lung injury and change macrophage phenotypes, although both old and young MSC-EVs have similar physical and phenotypical characteristics (Huang et al., 2019). Therefore, exploring the potential molecular mechanisms of MSC senescence is of great significance to the therapeutic application of MSCs in age-related diseases.

Since sirtuins were initially found to be related to the lifespan extension of yeast in 1997, the roles of sirtuin family members in cell longevity have been gradually revealed (Sinclair and Guarente, 1997). Seven members of sirtuin family are localized in different cellular compartments and highly conserved from bacteria to humans (Favero et al., 2015). As a mitochondria-localized deacetylase, Sirt3 had been linked to defense against oxidative stress in a variety of systems and their function executed depending on the content of intracellular NAD^+^ (Michan and Sinclair, 2007). Sirt3 participated in almost every major aspect of mitochondrial biology, such as mitochondrial respiratory, ATP synthesis, ROS production, and detoxification (Bause and Haigis, 2004). In particular, there were abundant evidences that Sirt3 was closely related to age-dependent elevation in mitochondrial oxidative stress (Lee et al., 2018). In Sirt3 heart-specific knockout (SIRT3^–^/^–^) mice, Sirt3 deficiency weakened resistance to oxidative stress, disrupted mitochondrial homeostasis, and finally resulted in obvious aging features in the myocardium, which suggested that Sirt3 might regulate age-related heart diseases by maintaining the normal biological functions of the mitochondria (Li et al., 2018). It has been reported that Sirt3 over-expression in late-passage MSCs reduced oxidative stress, enhanced their ability to differentiate, and thus ameliorated age-related senescence (Denu, 2017). Nonetheless, the mechanistic links between Sirt3 expression and oxidative stress relevant senescence are not well studied. The current study showed that Sirt3 mRNA and protein expressions were dramatically decreased in old and H_2_O_2_-pretreated MSCs compared to those in young MSCs, which indicated Sirt3 decline was associated with age-related elevated ROS. Despite the different triggering mechanisms, MSCs from natural aging or premature senescence models were accompanied by the accumulation of intracellular ROS. In this study, we tried to compare the role of Sirt3 under different activation mechanism of MSC senescence.

To further test the function of Sirt3 on ROS-related MSC senescence, we modulated its expression through gene manipulation. Senescent cells exhibited the changes in cellular morphology and gene levels, and SA-β-gal activity and the expression of the senescence-related factors p16^INK4A^ and p21^WAF1/CIP^ were commonly selected for senescence identification (Shibata et al., 2007; Wagner et al., 2008). Concomitant with elevated ROS concentration, an increased number of MSCs manifested as typically senescence-like morphological alterations, namely, increased cell body and vanishing stereoscopic sensation. Moreover, both MSCs from the old group and H_2_O_2_ addition group showed a high percentage of SA-β-gal-positive cells. Sirt3 supplementation in ROS-relevant senescent MSCs cannot only rejuvenate senescent appearance to a youthful phenotype but also decrease the number of SA-β-gal-positive cells. Molecular changes are also important biological indicators of cellular senescence. In Campisi and d’Adda di Fagagna (2007), summarized two classical effectors of senescence pathways, the p53-p21^WAF1/CIP^ and p16^INK4A^-pRB pathways. Subsequently, accumulating studies have clarified that p53-p21^WAF1/CIP^ and p16^INK4A^-pRB serve as significant pathways to regulate various cellular senescence (Stein et al., 1999). Although ample evidences suggest that p16^INK4A^ is more closely related to stress-related senescence, and p21^WAF1/CIP^ seems to be more associated with replicative senescence (Shang et al., 2016; He L. et al., 2017; He X. et al., 2017), there are still some different voices (Itahana et al., 2003; Brookes et al., 2004). Sugihara et al. (2018) demonstrated that H_2_O_2_ pretreatment in mesenchymal progenitor cells (MPCs) enhanced the expression of p21^WAF1/CIP^ but not that of p16^INK4A^, indicating that oxidative stress-related senescence can also be mediated by the up-regulation of p21^WAF1/CIP^. Our previous study has similar results that in MSC replicative senescence, p16^INK4A^ expression was elevated, while p21^WAF1/CIP^ expression was not significantly changed (data not shown). However, our current results showed both p16^INK4A^ and p21^WAF1/CIP^ expressions were significantly up-regulated in the old MSC group and H_2_O_2_-pretreated MSC group. We thus speculate that cell specificity might be the reason for the inconsistent expression of p16^INK4A^ and p21^WAF1/CIP^ in different types of senescent processes. We also analyzed the effects of Sirt3 over-expression on ROS-related senescent MSCs. Our findings showed that Sirt3 repletion in old or H_2_O_2_-pretreated MSCs suppressed the expression of p21^WAF1/CIP^ and p16^INK4A^ to some degrees, indicating that Sirt3 exerts the regulatory effects on cellular senescence in response to the elevated endogenous or exogenous oxidation levels. Additionally, we found that whether in the old or H_2_O_2_ group, Sirt3 supplement mediated by lentivirus markedly decreased the intracellular ROS content, further strengthening our finding that Sirt3 plays a regulatory role in oxidative stress-associated MSC senescence.

In mammals, there are three forms of SODs localized in different cellular compartments, defending cells from oxidative stress damage (Balaban et al., 2005). SOD2 is a specific SOD located in the mitochondria, which is considered as a scavenging enzyme that inhibits mitochondrial ROS (Liang et al., 2007). Ample evidences demonstrated that SOD2 might be a potential specific target of Sirt3, and enhanced Sirt3/SOD2 signaling improved endothelial reparative capacity of endothelial progenitor cells (EPCs) via suppressing mitochondrial oxidative stress (He et al., 2019). Chen et al. (2011) confirmed that Sirt3 over-expression can improve the antioxidant activity of SOD2 and enhance mitochondrial ROS-scavenging capacity. A study brought up that SOD2 might be directly activated by Sirt3 deacetylation at specific lysine residues (Qiu et al., 2010). Sirt3 can also enhance SOD2 activity by increasing FoxO3a DNA binding at the SOD2 promoter (Jacobs et al., 2008). All previous studies mentioned suggest that SOD2 may be an important target of Sirt3 in the process of cellular senescence. To test our hypothesis, we evaluated the expression and activity of SOD2. As expected, after Sirt3 over-expression, senescent MSCs presented young phenotypes. Meanwhile, intracellular SOD2 level was markedly elevated, as well as Sirt3 activity was obviously increased. These results imply that Sirt3 replenishment attenuating ROS-relevant MSC senescence is related to the Sirt3/SOD2 signaling pathway. To further clarify the possible mechanism of Sirt3/SOD2 axis regulating ROS-related MSC senescence, DNA damage and intracellular MDA levels were assessed. Gene silence of Sirt3 increased ROS production, and an excess of ROS damaged biological macromolecules, which can be reflected by comet assay and MDA content detection (Kim et al., 2010; Groschner et al., 2012; Ma et al., 2017). Xie et al. (2017) reported that Sirt3 attenuated cellular senescence in porcine fetal fibroblasts possibly via decreased oxidative damage and enhanced the SOD2 activity. In the present study, the expression and activity of Sirt3 and SOD2 were reduced in both senescent MSCs compared with young MSCs. Simultaneously, the intracellular ROS accumulation, DNA damage, and MDA contents were all elevated in senescent MSCs. However, after Sirt3 over-expression in senescent MSCs, the expression and activity of SOD2 increased, while the intracellular ROS level, DNA damage, and MDA levels decreased. Therefore, Sirt3 might inhibit MSC natural senescence and H_2_O_2_-induced premature senescence through facilitating SOD2 activation and alleviating ROS-induced injury. Our results were consistent with the published data showing that Sirt3 over-expression in senescent MSCs has protective antioxidant capacity, effectively eliminating DNA damage and lipid peroxidation, so as to alleviate the cellular senescence induced by ROS accumulation.

In conclusion, the results of the current study validated that Sirt3 has regulatory effects on ROS-relevant MSC senescence. Sirt3 over-expression in natural senescent and H_2_O_2_-induced premature senescent MSCs inhibited intracellular ROS generation and enhanced SOD2 levels and activity, thus reducing oxidative stress damage to delay cellular senescence. Our present study may not only enrich insights into the molecular mechanisms underlying stem cell senescence but also potentially provide a novel targeted therapeutic strategy for age-associated disorders. However, the complex mechanisms of Sirt3 regulating MSC senescence and its role *in vivo* merit in-depth exploration. Thus, further investigations are underway to unravel these issues to benefit therapeutic applications of MSCs.

## Materials and Methods

### Cell Culture

One to two-months-old (young group) and 15–18 months-old (old group) male Wistar rats were purchased from the Experimental Animal Center of Jilin University, Changchun, P.R. China. The whole bone marrow adherent method was used to isolate MSCs, as previously described (Ma et al., 2017). MSCs were cultured in complete medium containing 89% Dulbecco’s Modified Eagle Medium with nutrient mixture F-12 (DMEM-F12, Gibco, United States) supplemented with 10% fetal bovine serum (Gibco, United States) and 1% penicillin streptomycin (HyClone, United States). Then MSCs at passage 3 (P3MSCs) obtained by serial passages were used in subsequent experiments.

### H_2_O_2_ Treatment and Cell Proliferation Assay

MSCs (5 × 10^3^) were seeded in each well of 24-well plates with complete medium and cultivated for 24 h. For establishment of premature senescence model, MSCs were, respectively, treated with complete medium containing 50, 100, 200, and 400 μM H_2_O_2_ at 37°C with 5% CO_2_ for 2 h. Then, cells were washed twice with serum-free DMEM-F12 to remove residual H_2_O_2_ and cultured for additional 48 h to examine the sustained toxicity of H_2_O_2_ on cell proliferation. Cell Counting Kit-8 (CCK8, Dojindo, Japan) was used to determine cell survival according to the manufacturer’s instruction. Thereafter, the absorbance was measured at 450 nm using a microplate reader (TECAN, SWIT). The survival curve was drawn according to the absorbance value, and half lethal dose of H_2_O_2_ was calculated (Burova et al., 2013).

### Cell Growth Assay and Population Doubling Time

For cell growth assay, 5 × 10^3^ MSCs were seeded in each well of 24-well plates with complete medium. The cells were stained by trypan blue, and cell numbers were counted every day for 7 days. To detect population doubling time (PDT), 7 × 10^5^ MSCs were plated onto each 10 cm dish at t1, and the number of cells at this time was recorded as Nf. When cells reached 80% confluency, they were harvested and cell numbers (Ni) counted at this time (t2). PDT was calculated using the following formula: PDT = t2 − t1/ln (Nf/Ni)/ln (2).

### Cell Cycle Analysis

Cell cycle assay was performed using a Cell Cycle Detection Kit (KeyGEN BioTECH, China) according to the manufacturer’s instructions. In short, 1 × 10^6^ cells were collected and then fixed in 70% methanol overnight at 4°C. Cells were subsequently resuspended in phosphate-buffered saline (PBS) and incubated with 100 μl of RNaseA for 30 min at 37°C in the dark. Before flow cytometry analysis, 400 μl of propidium iodide (PI) was added at 4°C for at least 15 min. Cell cycle distribution was assessed using a FACS Calibur (BD Biosciences, United States) with Cell Quest software.

### Senescence-Associated-β-Galactosidase Activity Assay

The percentage of senescent cells was calculated to assess MSC senescence using a senescence cell histochemical staining kit (Beyotime, China) according to the manufacturer’s instructions. Briefly, cells were immobilized in fixation buffer for 15 min at room temperature, followed by washing twice with PBS. Then, 200 μl of staining solution mix was added before incubation for 12–14 h at 37°C. Ten high-power microscopic fields were randomly selected to count the number of blue cells (β-gal-positive cells) out of at least 200 cells.

### Apoptosis Assay

To measure cell apoptosis, PI-Annexin V Apoptosis Detection Kit I (BD Biosciences, United States) was used according to the manufacturer’s instructions. Briefly, MSCs were collected and washed three times. Then, cell pellets were resuspended in 1 × binding buffer (100 μl) and stained with Annexin V/FITC (5 μl) and PI (10 μl) for 15 min at room temperature in the dark. Afterward, apoptotic events were detected using a flow cytometer (FACS Calibur, BD Biosciences, United States).

### Measurement of Intracellular ROS

The intracellular accumulation of ROS was measured using a dihydroethidium (DHE) kit (Beyotime, China) according to the manufacturer’s instructions. Briefly, 1 × 10^5^ cells were seeded in 24-well plates and incubated with 10 μM DHE for 30 min at 37°C. Then, after removing the medium and washing the cells with serum-free culture medium, the fluorescence images were captured using fluorescence microscopy (excitation 300 nm and emission 610 nm) (OLYMPUS, Japan) and quantified with ImageJ software.

### Comet Assay

To determine DNA damage, comet assay was performed using a CometAssay Kit (Trevigen, United States) in accordance with the manufacturer’s instructions. Cells (4 × 10^3^) were collected and mixed with low-melting agarose and subsequently rapidly dripped onto a slide. After coagulation, the slide was placed in lysis buffer for 2 h at 4°C. The slides were then immersed in alkaline unwinding solution (pH > 13, 300 mM NaOH, and 1 mM EDTA) for 20 min at 4°C. Next, the slides were submerged with the mixture in a pre-cooled electrophoresis buffer (pH > 13, 300 mM NaOH, and 1 mM EDTA) and subjected to electrophoresis at 300 mA for 30 min. After being washed three times with PBS, cells were quickly incubated with PI staining and viewed under a fluorescence microscope. The olive tail moment (OTM) values were measured using CASP software.

### Real-Time Quantitative PCR Analysis

Total RNA was extracted from MSCs using TRIzol (Takara, China), and 1 μg of total RNA was used for cDNA synthesis using an RNA PCR Kit (AMV) Ver.3.0 (Takara, China). mRNA levels were determined by real-time quantitative polymerase chain reaction (RT-qPCR) using TransStart Top Green qPCR SuperMix (TRANS, China) in a 7300 Real-Time PCR System (ABI, United States). Relative gene expressions were normalized using β-actin mRNA as a reference and calculated using the 2^–ΔΔCt^ method. All the primers used in the experiment were designed and synthesized as shown in Table 1.

**TABLE 1:** The primers used in this study.

**Gene name**	**Forward (5′–3′)**	**Reverse (5′–3′)**
Sirt3	TGCACGGTCTGTCGAAGGTC	ATGTCAGGTTTCACA ACGCCAGT
SOD2	GAGCAAGGTCGCTTACAGA	CTCCCCAGTTGAT TACATTC
pl6^INK4A^	AACACTTTCGGTCGTACCC	GTCCTCGCAGT TCGAATC
p21^WAF1/CIP1^	GACATCACCAGGA TCGGACAT	GCAACGCTACTAC GCAAGTAG
β-actin	GGAGATTACTGCCCT GGCTCCTA	GACTCATCGTACTCCT GCTTGCTG

### Western Blot Analysis

MSCs were collected and incubated for 30 min on ice in RIPA Lysis Buffer (Beyotime, China) for extracting total protein. The BCA Protein Assay Kit (Beyotime, China) was used for protein quantification. Protein sample lysate (30 μg of each) was resolved by 10% sodium dodecyl sulfate (SDS)-polyacrylamide gels and then transferred onto PVDF membranes (Millipore, United States). Membranes were blocked at room temperature for 2 h with 5% non-fat milk in Tris-buffered saline (TBS) to avoid non-specific blots and then incubated with primary antibodies: anti-Sirt3 (1:1,000, Santa Cruz) and anti-SOD2 (1:1,000, Santa Cruz) overnight at 4°C. Membranes were washed three times to remove excessive primary antibodies and then incubated for 1 h at room temperature with anti-rabbit IgG secondary antibody at an appropriate dilution of 1:2,000. The immunoreactive protein bands were visualized on an electrochemiluminescence detection system (JENE, United Kingdom) by enhancing ECL Plus (Beyotime, China). β-Actin was used as an internal control.

### Measurement of SOD2 Enzyme Activity

SOD2 enzymatic activity was assayed using superoxide dismutase (SOD) assay kit with WST-1 (Nanjing Jiancheng, China). MSCs were adjusted to 1 × 10^6^ cell/ml after trypsinization, washed twice with PBS, and centrifuged at 1,000 rpm for 10 min. The supernatant was then removed. The precipitate obtained through centrifugation was crushed by ultrasonic wave, and the cell lysates were resuspended. According to the manufacturer’s instruction, SOD2 activity was determined with a microplate reader.

### Detection of Malondialdehyde Contents

The MDA was assayed according to Cell Malondialdehyde (MDA) Assay Kit-Colorimetric method (Nanjing Jiancheng, China). Briefly, the cell culture supernatant was discarded, and then, cells were scraped and transferred to the EP tube. The cell samples were prepared by addition of 0.5 ml extract and mixed for 2 min. Then, 0.1 ml of absolute ethanol and 1 ml of working solution were added and mixed. After incubation at 95°C for 40 min and centrifugation at 4,000 rpm for 10 min, the absorbance was measured by a microplate reader (Tecan, Switzerland) at 450 nm.

### Lentivirus Transduction of MSCs

The cells were transduced with lentiviral particles encoding rat Sirt3 or control vector as previously described (Pi et al., 2019). Briefly, cells were plated at 1.5 × 10^5^/well in six-well plate and incubated at 37°C for 18 h, and then, cells were transduced with lentivirus-expressing Sirt3 in the presence of 4 μg/ml polybrene (Genechem Co. Ltd., China) for 12 h. Sirt3 over-expressed MSCs were used in subsequent experiments.

### Statistical Analysis

All experimental data were expressed as the mean ± standard deviation (SD). Comparisons between two groups were performed using a two-tailed Student’s *t*-test. A *P* value < 0.05 was considered statistically significant.

## Data Availability Statement

The original contributions presented in the study are included in the article/supplementary material, further inquiries can be directed to the corresponding author.

## Ethics Statement

The animal study was reviewed and approved by the Ethics Committee of Jilin University.

## Author Contributions

CM and YS were responsible for performing the experiments and writing the manuscript. CP, HW, HS, XY, and YS contributed to data collection, data analysis and interpretation. XH was responsible for conception, design, manuscript revision and confirmation, and financial support. All authors have read and approved the final version of the manuscript.

## Conflict of Interest

The authors declare that the research was conducted in the absence of any commercial or financial relationships that could be construed as a potential conflict of interest.
